# Complete Chloroplast Genomes and Comparative Analyses of Three *Paraphalaenopsis* (Aeridinae, Orchidaceae) Species

**DOI:** 10.3390/ijms241311167

**Published:** 2023-07-06

**Authors:** Jinliao Chen, Fei Wang, Zhuang Zhao, Minghe Li, Zhongjian Liu, Donghui Peng

**Affiliations:** Key Laboratory of National Forestry and Grassland Administration for Orchid Conservation and Utilization at Landscape Architecture and Arts, Fujian Agriculture and Forestry University, Fuzhou 350002, China; fjchenjl@126.com (J.C.);

**Keywords:** *Paraphalaenopsis*, Aeridinae, chloroplast genome, phylogenetic analysis

## Abstract

*Paraphalaenopsis*, a genus of perennial herbs from the family Orchidaceae, contains a number of ornamental species. However, there is no information on the chloroplast genomes of *Paraphalaenopsis*, which limits our studies of this genus. In this study, we reported the chloroplast genomes of three species of *Paraphalaenopsis* (*P. labukensis*, *P. denevel*, and *P. laycockii* ‘Semi-alba’) and performed comprehensive comparative analysis. These three chloroplast genomes showed a typical quadripartile structure. Their lengths ranged from 147,311 bp to 149,240 bp. Each genome contained 120 unique genes, including 74 protein-coding genes, 38 tRNA genes, and 8 rRNA genes. Comparative analysis revealed major differences in sequence divergence in the three chloroplast genomes. In addition, six hypervariable regions were identified (*psbM-trnD^GUC^*, *psbB*, *ccsA*, *trnK^UUU^*, *trnS^GCU^-trnG^UCC^*, *rps16-trnQ^UUG^*) that can be used as DNA molecular markers. Phylogenetic relationships were determined using the chloroplast genomes of 28 species from 12 genera of Aeridinae. Results suggested that *Paraphalaenopsis* was a clade of Aeridinae that was sister to the *Holcoglossum-Vanda* clade, with 100% bootstrap support within Aeridinae. The findings of this study provided the foundation for future studies on the phylogenetic analysis of Aeridinae.

## 1. Introduction

*Paraphalaenopsis* belongs to the tribe of Vandeae, a subtribe of Aeridinae, of the family Orchidaceae. *Paraphalaenopsis* is endemic to Borneo (Kalimantan, Sarawak, and Sabah) and is related to *Renanthera*, *Aerides*, *Doritis*, *Phalaenopsis*, and *Kingidium* [1]. This genus consists of four species, including *P. labukensis*, *P. laycockii*, *P. serpentilingua*, and *P. denevei*. *Paraphalaenopsis* is an epiphytic herb, and the leaves are terete or nearly terete and hang naturally, such as a pencil or rat-tail, known as the “rat-tailed phalaenopsis” [2]. The flowers of *Paraphalaenopsis* species usually release a strong scent analogous to cinnamon or ripe bananas [2]. However, only a few reports have been documented about *Paraphalaenopsis*. Considering that the species of this genus are morphologically similar, precise species recognition based on molecular markers is particularly important for the rational utilization of this genus of plants. 

Moreover, some researchers have used molecular methods to explore phylogenetic relationships within the genus *Paraphalaenopsis* and its phylogenetic position in the family Aeridinae, while the selected DNA fragments are one-sided and partially complete, with low bootstrap support values, which imposes certain limitations on the phylogenetics of *Paraphalaenopsis* [3,4]. Therefore, it is necessary to further explore the phylogeny of *Paraphalaenopsis* species within Aeridinae.

Due to its short length, large number of gene copies, highly conserved sequence, and low genetic recombination rate, the chloroplast genome is an ideal tool for studying genetic differences and molecular phylogeny among species [5,6,7]. In recent years, as more and more chloroplast genomes have been reported, research on plant phylogeny based on chloroplast genomes has provided effective solutions to the systematic problems of some difficult taxa [6,8,9,10,11,12]. Recently, Li et al. [11] reported the phylogenetic relationships of the chloroplast genomes of 12 *Holcoglossum* species, and Xiao et al. [10] reported the phylogenetic relationships of the chloroplast genomes of four *Renanthera* species, providing a wealth of chloroplast genome resources for the study of Aeridinae plants. Unfortunately, there have been no reports on the chloroplast genomes of *Paraphalaenopsis*.

In this study, we presented the whole chloroplast genome sequence of *Paraphalaenopsis* and investigated the utility of these new genomic resources and their relationships with other Aeridinae species. We analyzed the structural features and sequence divergence of the chloroplast genomes in *Paraphalaenopsis* and performed plastome-based analyses, comparing the differences among selected closely related species. Finally, we inferred the phylogenetic relationships of *Paraphalaenopsis* within Aeridinae based on the complete chloroplast genome sequence.

## 2. Results

### 2.1. Genome Characteristic

**Figure 1:**
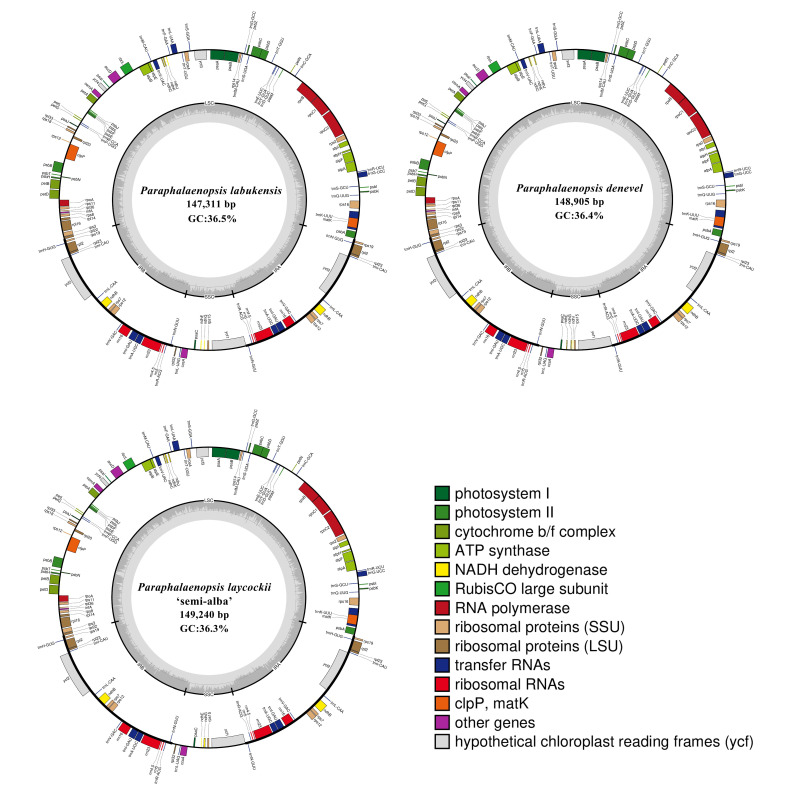
Chloroplastic genome structure of three *Paraphalaenopsis* species (*P. labukensis*, *P. denevel*, *P. laycockii* ‘Semi-alba’).

In this study, the complete chloroplasts of three *Paraphalaenopsis* species were obtained for the first time, with genome sizes ranging from 147,311 bp (*P. labukensis*) to 149,240 bp (*P. laycockii* ‘Semi-alba’) (Figure 1). Three chloroplast genomes of *Paraphalaenopsis* exhibited the quadripartite structure typical of most angiosperms, consisting of two copies of IR regions (24,915–25,412 bp), a large single-copy region (LSC, 85,989–86,761 bp), and a small single-copy region (SSC, 11,492–11,655 bp) (Table 1). The G/C content was approximately 36.4% (Table 1), which is comparable to other previously sequenced chloroplast genomes of Orchidaceae [13,14]. The GC content of each region varied in the three chloroplast genomes and was (43.1–43.3%), (27.5–27.8%), and (33.4–33.7%) for the IR, SSC, and LSC regions, respectively (Table 1). 

The chloroplast genomes of *Paraphalaenopsis* encoded 120 genes (including repetitive genes), consisting of 74 protein-coding genes, 38 transfer RNA (tRNA) genes, and eight ribosomal RNA (rRNA) genes (Table 1). Functional *ndh* genes are lost or pseudogenized in all *Paraphalaenopsis* species. The ndh genes were all pseudogenes with 6–7 members in each plastome (Table 1). The plastomes of *P. denevel* possessed seven (*ndhB*/*C*/*E*/*G*/*I*/*J*/*K*) pseudogenes; *P. labukensis* and *P. laycockii* ‘semi-alba’ possessed six (*ndhB*/*C*/*E*/*G*/*J*/*K*) pseudogenes, respectively (Table 2). Most genes of the three chloroplast genomes appeared as a single copy in the LSC or SSC region, with 19 gene duplications in the IR regions; six tRNA genes and six protein-coding genes contained one intron, and three genes (*ycf3*, *clpP*, and *rps12*) contained two introns (Table 2).

**Figure 2:**
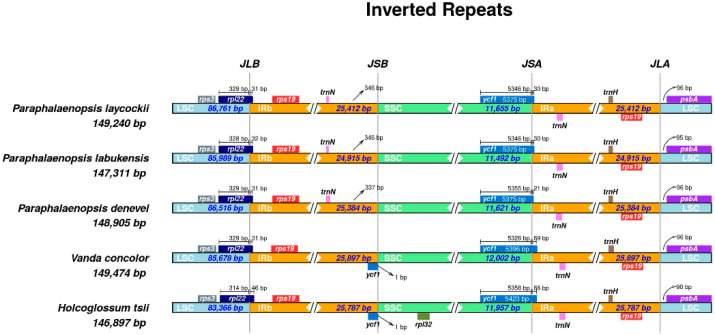
Comparison of connections between LSC, SSC, and IR regions. *P. labukensis*, *P. denevel*, *P. laycockii* ‘Semi-alba’, *Vanda concolor*, and *Holcoglossum tsii* chloroplast genomes.

We comprehensively compared the positions of IR boundaries and adjacent genes in three *Paraphalaenopsis* and two other closely related orchid species (Figure 2). Although the length of IR regions varied less among the five species, there were some differences in IR expansions and contractions. The *trnN-ycf1* genes were located at the crossing points of the SSC/IRa (JSA) regions. The *ycf1* gene was duplicated in two other Aeridinae species—*Vanda concolor* and *Holcoglossum tsii*, which were located at the IRb/SSC (JSB) boundary—but not in the *Paraphalaenopsis* species. The *rpl22 -rps19- psbA* were located at the intersections of the LSC/IR regions. The *rpl22* genes of LSC crossed with IRb in the chloroplast genomes of five species, with the length ranging from 31 bp to 46 bp. The *psbA* gene was complete in the LSC region in all these chloroplast genomes, 90–96 bp from the IRa/LSC (JLA) boundary. Moreover, the *trnN* and *rps19* genes were completely in the IR regions and duplicated in the chloroplast genomes of *Paraphalaenopsis*.

### 2.2. Repeat and SSR Analysis

**Figure 3:**
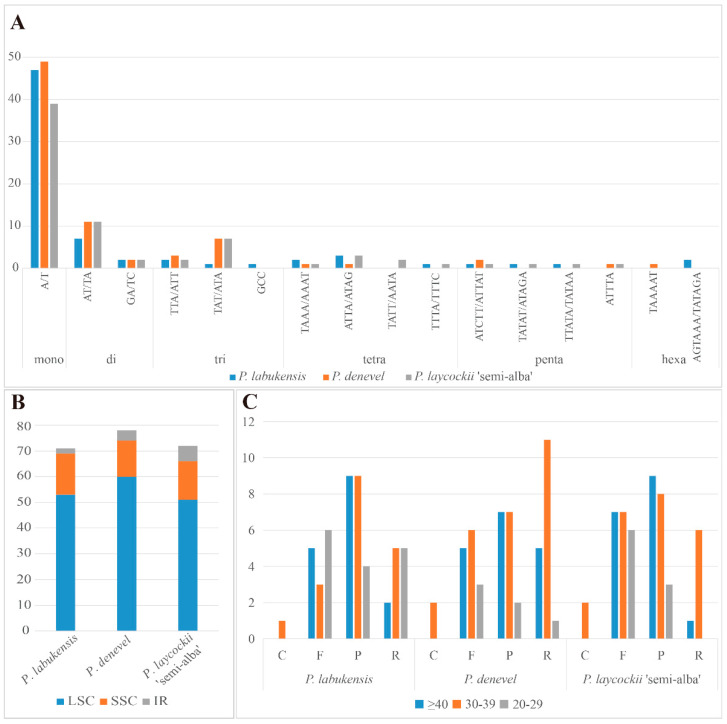
Analysis of simple sequence repeats (SSRs) and repeated sequences in the chloroplast genomes of *P. labukensis*, *P. denevel*, and *P. laycockii* ‘Semi-alba’. (**A**) Type and number of each identified SSR; (**B**) Number of SSRs for each *Paraphalaenopsis* species by location in IR, LSC, and SSC; (**C**) Total of three species with four repeat types.

*Paraphalaenopsis* species had a total of 71 (*P. labukensis*)–78 (*P. denevel*) SSRs (Figure 3A, Supplementary Table S3). Among the SSRs, mononucleotide repeats were the most abundant. At least 39–49 mononucleotide repeats were found in the three *Paraphalaenopsis* species: 9–13 were dinucleotide repeats, 4–10 were trinucleotide repeats, 2–7 were tetranucleotide repeats, and 1–2 were pentanucleotide repeats. Hexanucleotide repeats were 1–2 repeats in all the species except *P. laycockii* ‘Semi-alba’, which had no repeats. Most mononucleotides and dinucleotides consisted of A/T and AT/AT (Figure 3A, Supplementary Table S3). Most SSRs were located in the LSC region, while a few were located in the IR region. (Figure 3B, Supplementary Table S3). 

Four types of repeats (completement, forward, palindrome, and reverse) were analyzed in the chloroplast genomes of three *Paraphalaenopsis* species. Each genome contained 49 large repeats (>20 bp); almost all repeats were in the range of >30 bp in length, with the fewest in the range of 20–29 bp. Of these, 1–2 were complement (C), 14–20 were forward (F), 16–22 were palindromic (P), and 7–17 were reverse (R) (Figure 3C, Supplementary Table S2).

### 2.3. Comparative Genomic Divergence and Genome Rearrangement

**Figure 4:**
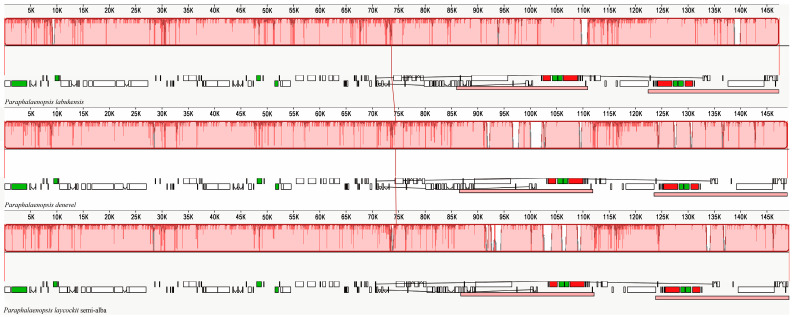
Chloroplast genomes comparison of three species of *Paraphalaenopsis* using a progressive MAUVE algorithm.

**Figure 5:**
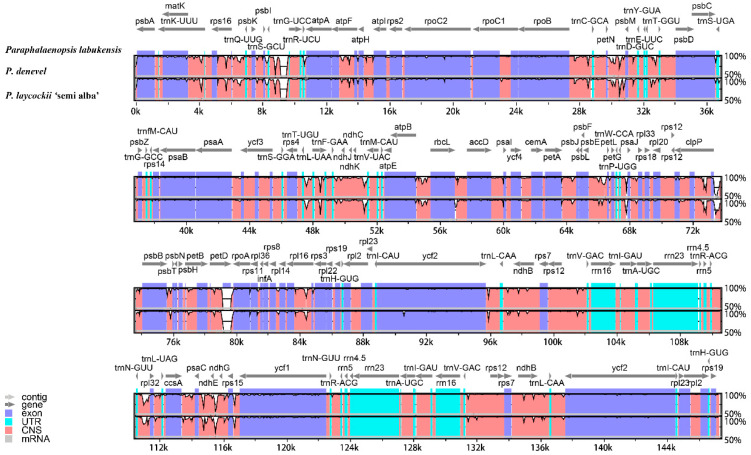
Global alignment of three *Paraphalaenopsis* chloroplast genomes using mVISTA with *P. labukensis* as reference. The y-axis shows the coordinates between the chloroplast genomes.

Comparative and collinearity analyses of chloroplast genomes can reveal differences between species. We found that the three chloroplast genome sequences of *Paraphalaenopsis* have a high degree of similarity, and no restructuring occurred. (Figure 4). Sequence differences exist in several regions, including *trnK^UUU^*, *trnS^GCU^-trnR^UCU^*, *petN-psbM*, *psbE-petL*, *clpP-psbB*, *petD*, *psaC-ndhE*, *rbcL-accD*, *ycf2*, *rpl16*, and *ndhB* of the three *Paraphalaenopsis* species (Figure 5).

**Figure 6:**
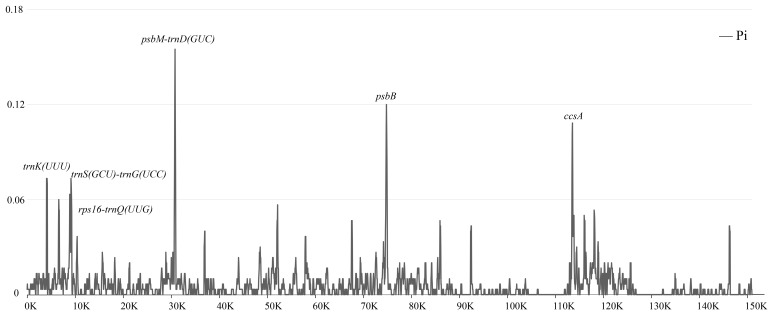
Sliding window test of nucleotide diversity (Pi) in the *Paraphalaenopsis* chloroplast genomes. Window length: 600 bp; step size: 200 bp. X-axis: the position of the midpoint of a window. Y-axis: nucleotide diversity of each window.

To further analyze the mutation hotspots of the chloroplast genomes of *Paraphalaenopsis* species, we used DnaSP6 to analyze the nucleotide diversity (Pi) for the alignment of the complete genomes (Figure 6, Supplementary Table S4). The nucleotide diversity (Pi) values of the three chloroplast genomes ranged from 0 to 0.155, and sliding window analysis showed that mutation hotspots included *psbM-trnD^GUC^*, *psbB*,*ccsA*, *trnK^UUU^*, *trnS^GCU^-trnG^UCC^*, and *rps16-trnQ^UUG^*, which had higher Pi values (>0.06) in the LSC and SSC regions. These six mutational hotspots may contain information about more rapidly evolving sites and could be potential molecular markers.

### 2.4. Phylogenetic Analysis

**Figure 7:**
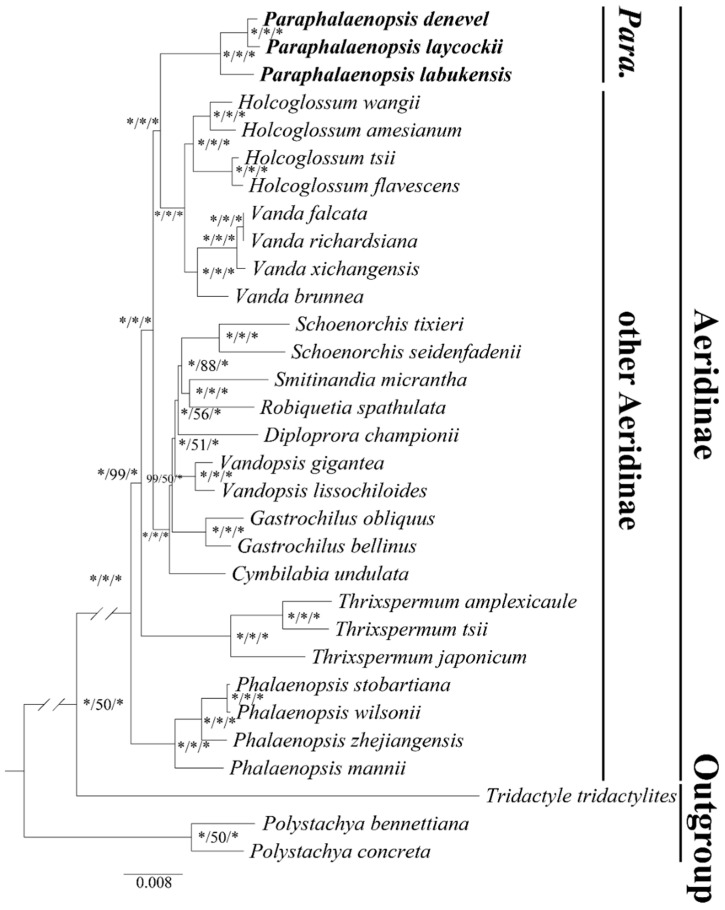
Phylogenetic tree of *Paraphalaenopsis* and other 24 Aeridinae species based on the complete chloroplast genome data. Numbers near the nodes are bootstrap percentages and Bayesian posterior probabilities (BSML left, BSMP middle, and PP right).-indicates that a node is inconsistent between the topology of the MP/ML trees and the Bayesian tree. * indicates that the node has 100 bootstrap percentage or 1.00 posterior probability.

We inferred the phylogenetic relationships of *Paraphalaenopsis* species and other Aeridinae species by ML analysis (IQ-tree ultrafast method) of complete chloroplast genomes and 68 protein-coding genes, resulting in two trees with the same topology. (Figure 7; Supplementary Figure S1). All the branch nodes in the phylogenetic tree were strongly supported in the ML analysis and the BI analysis (BS ≥ 75%, PP ≥ 0.90). All *Paraphalaenopsis* species formed a monophyletic subclade in both trees. 

## 3. Discussion

In this study, we obtained the chloroplast genome sequences of three species of *Paraphalaenopsis* using next-generation sequencing technology. The chloroplast genomes had a typical tetrad structure and a size range of 147,311 bp to 148,905 bp, wherein the structure and gene order were highly conserved, in line with the range of previously reported orchid chloroplast genomes [6,10,11,12]. These results suggest that the chloroplast genomes are still relatively conserved in *Paraphalaenopsis*. In addition, a total of 71–78 SSRs were detected in the three chloroplast genomes, of which 39–49 were mononucleotide repeats. Most of the SSR sequences are often composed of A/T or AT/AT, a phenomenon that has also been observed in other plant species [6,15,16]. With abundant SSR loci associated with polymorphisms in the chloroplast genomes of different species, they are often used as molecular markers for species identification [17,18,19].

The variation, contraction, and expansion of the IR regions are common phenomena in the evolution process of angiosperms [20]. These phenomena may occur at the border of inverted repeats (IRs) and single-copy regions (LSC and SSC), allowing certain genes into IR or SC regions [21]. We observed that the ycf1 gene in the SSC region of *Vanda concolor* and *Holcoglossum tsii* extended across the JSA into the IRA region. This situation did not appear in the three *Paraphalaenopsis* species, and the length of their IR regions ranged from 24,915 bp to 25,412 bp, which was no significant difference. This suggests that the *Paraphalaenopsis* species did not undergo significant expansion/contraction in the IR regions.

Nucleotide diversity (Pi) can indicate the degree of variation of nucleic acid sequences in different species, and the position with higher variability can be used as a molecular marker of population genetics [22,23]. Chloroplast genome mutation hotspots are convenient and practical methods for developing DNA barcodes, which have been demonstrated in orchids [8,9,24,25,26,27]. In this study, using comparative chloroplast genomics analysis, we compared the complete chloroplast genomes and DNA sequence polymorphisms based on mVISTA and DnaSP v6.0. We observed that noncoding regions of *Paraphalaenopsis* chloroplast genomes exhibited higher polymorphism than coding regions, which is similar to most plants. In addition, most regions except the IR regions had high Pi values, indicating that these regions have the potential to design molecular markers. We propose that six hypervariable regions, *psbM-trnD^GUC^*, *psbB*, *ccsA*, *trnK^UUU^*, *trnS^GCU^-trnG^UCC^*, and *rps16-trnQ^UUG^*, can be used as potential molecular markers for the identification of *Paraphalaenopsis.*

Chloroplast genomes are highly conserved and have been widely applied in phylogenetic and evolutionary studies, which play a vital role in species identification [8,9,13,14,28]. We analyzed the phylogenetic relationships of *Paraphalaenopsis* belonging to Aeridinae by using the complete chloroplast genome sequences. In the unilateral analysis based on chloroplast genomes, *Paraphalaenopsis* and *Holcoglossum-Vanda* were sister groups and belonged to the Aeridinae [4]. This is consistent with the results of traditional classification and short gene sequence studies [3,4]. However, these results are restricted because of the maternal inheritance of the chloroplast genome [19,29], and accurate phylogenetic relationships still require a comprehensive analysis of nuclear and organellar genes [14,30]. In addition, of the 85 genera of Aeridinae, only 20 genera have been sequenced so far. In the future, further genome sequencing will be required to determine the relationships between *Paraphalaenopsis* and other species of the subtribe Aeridinae.

## 4. Materials and Methods

### 4.1. Plant Materials, DNA Extraction and Sequencing

Three *Paraphalaenopsis* species were selected, including *P. labukensis*, *P. denevel* and *P. laycockii* ‘Semi-alba’. *P. labukensis* and *P. denevel* were introduced and cultivated in the Shanghai Chen Shan Botanical Garden, Shanghai Province, China. *P. laycockii* ‘Semi-alba’ was introduced and cultivated in the China National Botanical Garden, Beijing Province, China. As shown in Supplementary Table S1, their voucher information was provided. The total DNA of leaf samples was extracted using the CTAB method [31]. Short-insert (500 bp) pair-end (PE) libraries were constructed, and the sequencing was performed by the Beijing Genomics Institute (Shenzhen, China) on the Illumina HiSeq 2500 platform with a read length of 150 bp. At least 10 Gb of clean data were obtained for each species.

### 4.2. Chloroplast Genome Assembly and Annotation

Chloroplast genome assembly and annotation were performed following previously described methods [32]. In short, the paired-end reads were assembled using the GetOrganelle pipeline (https://github.com/Kinggerm/GetOrganelle, accessed on 5 May 2023). Then the filtered reads were assembled using SPAdes version 3.10 [33]. The published chloroplast genome of *Phalaenopsis hygrochila* (MN124430) was chosen as a reference genome for assembling chloroplast genomes. Gene annotation was carried out using DOGMA [34] and checked with Geneious Prime v2021.1.1 [35]. The circle maps were drawn using OGDRAW [36].

### 4.3. Genome Comparison and Analysis, IR Border and Divergence Analyses 

The chloroplast genomes of three *Paraphalaenopsis* species were aligned with mVISTA using the alignment program LAGAN [37], using the sequence of *P. labukensis* as a reference. Rearrangements of chloroplast genomes were detected and graphed using Mauve in three species [38]. The boundaries between the IRs, SSCs, and LSCs of the chloroplast genomes were compared using the online program IRscope (https://irscope.shinyapps.io/irapp, accessed on 5 May 2023) [39].

To identify the mutational hotspot regions and genes, the chloroplast genome sequences were aligned using MAFFT v7 [40]. Then, the nucleotide diversity (Pi) of three chloroplast genomes of *Paraphalaenopsis* was calculated using DnaSP v6.12.03 (DNA sequence polymorphism) [41]. Highly mutated hotspot regions were identified by a sliding window strategy. The step size was set at 200 bp, with a 600 bp.

### 4.4. Repeat Sequence Analysis

The online software REPuter (https://bibiserv.cebitec.uni-bielefeld.de/reputer, accessed on 5 May 2023)was used to identify the repeat sequences, including forward, palindrome, reverse, and complementary long repeats [42]. The maximum and minimum repeat sizes were set to 50 bp and 20 bp, respectively, while the Hamming distance was set to 3. MISA-web was used to detect simple sequence repeats (SSRs). The thresholds for mono-, di-, tri-, tetra-, penta-, and hexa-nucleotide SSRs and the minimum number of repeats were set to 10, 5, 4, 3, 3, and 3, respectively [43].

### 4.5. Phylogenetic Reconstruction

We used the whole chloroplast genomes and 68 protein-coding sequences to perform the phylogenetic analysis of 30 species of Orchidaceae. Three species from *Polystachya* (*P. bennettiana* and *P. concreta*) and *Tridactyle* (*T. tridactylites*) were used as outgroups. Of these 30 species, three *Paraphalaenopsis* species are newly sequenced, and the other 27 species of 13 genera are from the complete plastid data publicly available at the National Center for Biotechnology Information (NCBI). A list of the taxa analyzed with voucher information and GenBank accessions is provided in Supplementary Table S1. The whole chloroplast genome sequences were aligned by Geneious Prime v2021.1.1 [18]. A total of 68 protein-coding genes were aligned by PhyloSuite v1.2.2 [44]. Phylogenetic relationships were analyzed by using maximum parsimony (MP), maximum likelihood (ML), and Bayesian inference (BI) on the CIPRES Science Gateway website [45]. All characters were equally weighted and unordered, and a heuristic search was performed using 1000 random sequence repeats and TBR branch swapping. For the analysis of ML, the GTRCAT model was specified for all datasets, and self-expanding analyses with 1000 repetitions were performed [46]. Bayesian analysis was performed using MrBayes v. 3.2.6 [47], and four Markov chains were run for 10,000,000 generations, sampling one tree every 100 generations. The first 25% of the trees were discarded as burn-in samples to ensure that each chain reached a steady state and the estimated posterior probabilities (PP).

## 5. Conclusions

In the present study, three chloroplast genomes of *Paraphalaenopsis* were first sequenced and assembled, whose structural features were similar to those of most species of Orchidaceae. Only the genome size, GC content, repeats, and IR boundaries showed certain differences, and all *ndh* genes were entirely lost or pseudogenic in plastids. This provides clues for understanding the interspecific diversity among *Paraphalaenopsis* chloroplast genomes. In addition, six hypervariable regions were identified that can be used as molecular markers to identify *Paraphalaenopsis*. The results not only enrich the Orchidaceae chloroplast genome data but also provide a certain theoretical basis for the phylogenetic reconstruction of Aeridinae.

## Figures and Tables

**Table 1:** Characteristics of the complete chloroplast genomes of *Paraphalaenopsis* strains.

Species	Size (bp)	LSC (bp)	SSC (bp)	IRs (bp)	Number of Genes	Protein Coding Genes	tRNA Genes	rRNA Genes	Total GC (%)	LSC GC (%)	SSCGC (%)	IR GC (%)	The Number of *ndh* Gene Loss /Pseudogenization
*P. labukensis*	147,311	85,989	11,492	24,915	120	74	38	8	36.5	33.7	27.8	43.3	7 (5)
*P. denevel*	148,905	86,516	11,621	25,384	120	74	38	8	36.4	33.5	27.5	43.2	8 (4)
*P. laycockii* ‘semi-alba’	149,240	86,761	11,655	25,412	120	74	38	8	36.3	33.4	27.6	43.1	7 (5)

**Table 2:** The list of genes in the chloroplast genomes of *Paraphalaenopsis* species.

Classfication	Genes
Genetic apparatus	
Large ribosomal subunits	*rpl2*(×2)a, *rpl14*, *rpl16*a, *rpl20*, *rpl22*, *rpl23*(×2), *rpl32*, *rpl33*, *rpl36*
Small ribosomal subunits	*rps2*, *rps3*, *rps4*, *rps7*(×2), *rps8*, *rps11*, *rps12*(×2) ^b^, *rps14*, *rps15*, *rps16*a, *rps18*, *rps19*(×2)
RNA polymerase subunits	*arpoA*, *rpoB*, *rpoC1*, *rpoC2*
Other genes	*accD*, *infA*, *ccsA*, *clpPb*, *matK*
Ribosomal RNAs	*rrn4.5*(×2), *rrn5*(×2), *rrn16*(×2), *rrn23*(×2)
Transfer RNAs	*trnA-*UGC(×2)a, *trnC-*GCA, *trnD-*GUC, *trnE-*UUC, *trnF-*GAA, *trnG-*GCC, *trnG-*UCC ^a^, *trnH-*GUG(×2), *trnI-*CAU(×2), *trnI-*GAU(×2) ^a^, *trnK-*UUU ^a^, *trnL-*CAA(×2), *trnL-*UAA a, *trnL-UAG*, *trnM-*CAU, *trnN-*GUU(×2), *trnP-*UGG, *trnQ-*UUG, *trnR-*ACG(×2), *trnR-*UCU, *trnS-*GCU, *trnS-*GGA, *trnS-*UGA, *trnT-*UGU, *trnT-*GGU, *trnV-*GAC(×2), *trnV-*UAC a, *trnW-*CCA, *trnY-*GUA, *trnfM-*CAU
Light dependent photosynthesis	
Photosystem I	*psaA*, *psaB*, *psaC*, *psaI*, *psaJ*
Photosystem II	*psbA*, *psbB*, *psbC*, *psbD*, *psbE*, *psbF*, *psbH*, *psbI*, *psbJ*, *psbK*, *psbL*, *psbM*, *psbN*, *psbT*, *psbZ*
NAD(P)H dehydrogenase complex	*ndhJ*, *ndhK*, *ndhC*, *ndhB*(×2), *ndhE*, *ndhG*, *ndhI*^c^
F-type ATP synthase	*atpA*, *atpB*, *atpF*a, *atpE*, *atpH*, *atpI*
Cytochrome b/f complex	*petA*, *petB*a, *petD*a, *petG*, *petL*, *petN*
Light independent photosynthesis	
Large subunit ofRubisco	*rbcL*
Function uncertain	*ycf1*, *ycf2*(*×2*), *ycf3*^b^, *ycf4*

^a^ Gene with one intron; ^b^ Gene with two introns; ^c^ Gene lost in *P. labukensis* and *P. laycockii* ‘Semi-alba’; (×2) Gene with two copies.

## Data Availability

The three chloroplast genome sequences of *Paraphalaenopsis* are deposited in GenBank of the National Center for Biotechnology Information (NCBI) repository, accession numbers OR159902 to OR159904.

## Supplementary Materials

The following supporting information can be downloaded at: https://www.mdpi.com/article/10.3390/ijms241311167/s1.
