# Supplementary material for: Complete Chloroplast Genomes and Comparative Analyses of Three Paraphalaenopsis (Aeridinae, Orchidaceae) Species
Source: Int J Mol Sci. 2023 Jul 6;24(13):11167. doi: 10.3390/ijms241311167 (PMC10342943; doi:10.3390/ijms241311167)
Supplement: Supplementary file 1 [file ijms-24-11167-s001.zip › Supplementary Figures and Tables.pdf]

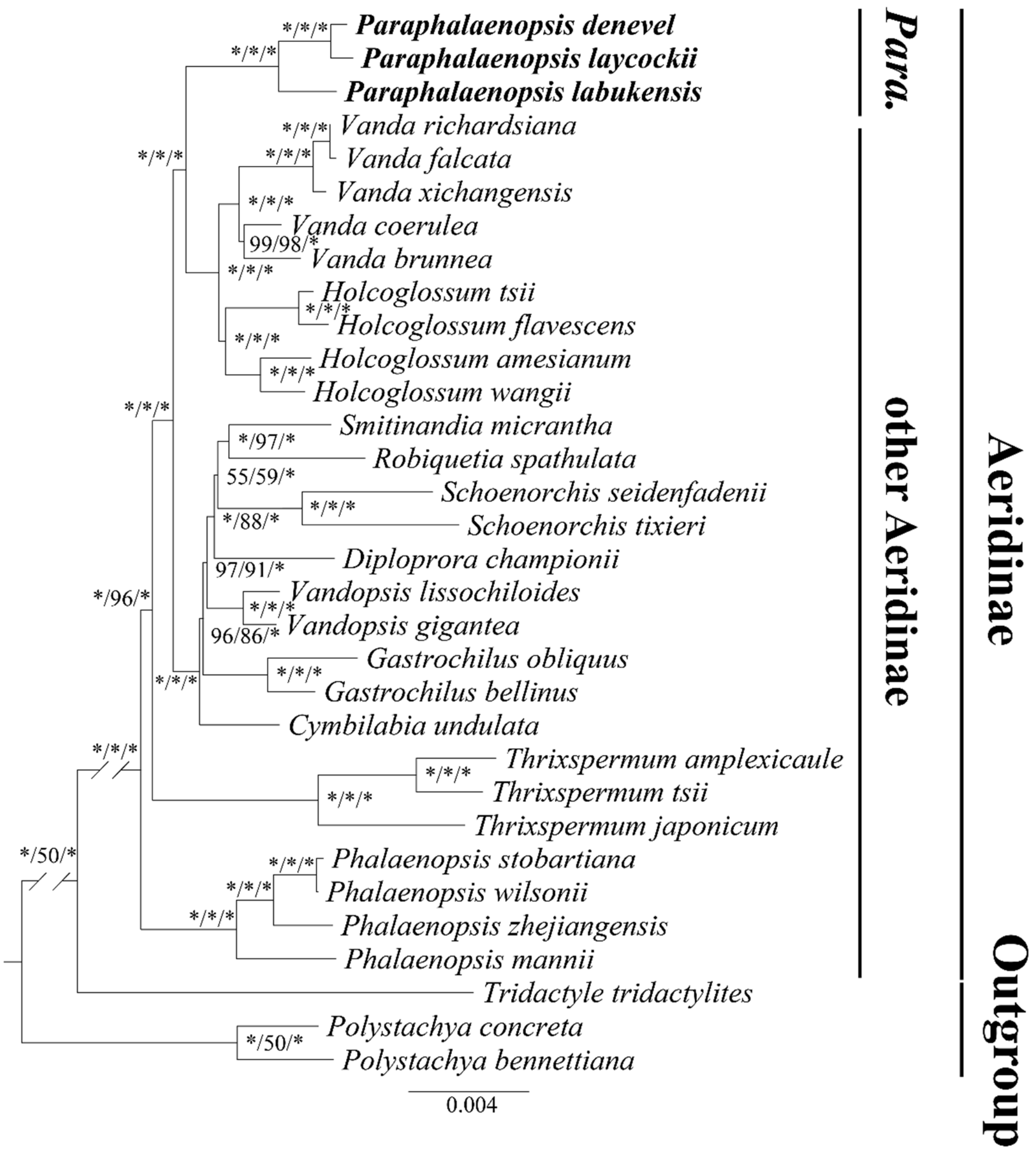

Supplementary Figure S1. Phylogenetic analysis of 28 Aeridinae species based on 68 protein coding genes. Numbers near the nodes are bootstrap percentages and Bayesian posterior probabilities (BSML left, BSMP middle, and PP right). - indicates that a node is inconsistent between the topology of the MP/ML trees and the Bayesian tree. * indicates that the node is 100 bootstrap percentage or 1.00 posterior probability.

Supplementary Table S1 Source and voucher information for this study. *P. labukensis* and *P. denevel* were introduced and cultivated in Shanghai Chen Shan Botanical Garden, Shanghai province, China. *P. laycockii* 'semi-alba' was introduced and cultivated in the China National Botanical Garden, Beijing province, China. And other voucher specimens were deposited in the herbariums of National Center for Biotechnology Information (NCBI).

Species	Source	Voucher	GenBank accession numbers
Paraphalaenopsis denevel	This study		OR159903
Paraphalaenopsis labukensis	This study		OR159902
Paraphalaenopsis laycockii 'semi-alba'	This study		OR159904
Holcoglossum wangii	Li et al., 2019	13881	NC041520
Holcoglossum amesianum	Li et al., 2019	9419	NC041511
Holcoglossum tsii	Chen et al., 2020	LDKAe67	NC048459
Holcoglossum flavescens	Li et al., 2019	8943	NC041512
Vanda falcata	Kim et al., 2017	PDBK 2014-0010	KT726907
Vanda richardsiana	Kim et al., 2017	PDBK 2014-0945	KT726908
Vanda xichangensis	Liu et al., 2019	Z.J. Liu 2747	NC047197
Vanda brunnea	Li et al., 2019	13059	NC041522
Schoenorchis tixieri	Liu et al., 2020	Liu4123	MN124407
Schoenorchis seidenfadenii	Liu et al., 2020	Liu5230	MN124434
Smitinandia micrantha	Liu et al., 2020	Liu21119	MN124406
Robiquetia spathulata	Liu et al., 2020	Liu5248	MN124410
Diploprora championii	Liu et al., 2020	Liu4480	MN124409
Vandopsis gisantea	Liu et al., 2020	Liu7990	MN124403
Vandopsis lissochiloides	Liu et al., 2020	Lior070	MN124443
Gastrochilus obliquus	Liu et al., 2020	Liu4191	MN124429
Gastrochilus bellinus	Liu et al., 2020	Lior011	MN124427
Cymbilabia undulata	Liu et al., 2020	Lior078	MN124402
Phalaenopsis stobartiana	Zhou et al., 2021	-	NC059917
Phalaenopsis wilsonii	Wang et al., 2021	-	NC057488
Phalaenopsis zhejiangensis	Jiang et al., 2021	-	MZ326749
Phalaenopsis mannii	Zhou et al., 2020	HDL-YN2019-12A	NC050940
**Outgroup**			
Tridactyle tridactylites	D'haijère et al., 2022	-	MW760855
Polystachaya bennettiana	Jiang et al., 2022	-	NC066148
Polystachaya concreta	Jiang et al., 2022	-	NC066150

Supplementary Table S2. The details information of long repeats.

seq len	species	*P. labukensis*	*P. denevel*	*P. laycockii* 'semi-alba'
⩾40	Complement	0	0	0
	Forward	5	5	7
	Palindrome	9	7	9
	Reverse	2	5	1
30-39	Complement	1	2	2
	Forward	3	6	7
	Palindrome	9	7	8
	Reverse	5	11	6
20-29	Complement	0	0	0
	Forward	6	3	6
	Palindrome	4	2	3
	Reverse	5	1	0

Supplementary Table S3. The details information Small simple repeats.

species	mono	di	tri	tetra	penta	hexa	total
P. laycockii 'semi-alba'	39	13	9	7	4	0	72
P. denevel	49	13	10	2	3	1	78
P. labukensis	47	9	4	6	3	2	71

species	LSC	SSC	IR	total
P. laycockii 'semi-alba'	51	15	6	72
P. denevel	60	14	4	78
P. labukensis	53	16	2	71

Supplementary Table S4. The nucleotide diversity of three *Paraphalaenopsis* Chloroplast genomes.

Sliding window test of nucleotide diversity (Pi) in the Paraphalaenopsis chloroplast genomes. Win-dow length: 600 bp; step size: 200 bp.
